# Effects of interleukin-1β and tumor necrosis factor-α on macrophage inflammatory protein-3α production in synovial fibroblast-like cells from human temporomandibular joints

**DOI:** 10.1111/jop.12040

**Published:** 2013-01-18

**Authors:** Miwa Akutsu, Naomi Ogura, Ko Ito, Mutsumi Kawashima, Tsuyoshi Kishida, Toshirou Kondoh

**Affiliations:** 1 Department of Maxillofacial Surgery, Nihon University School of Dentistry at MatsudoMatsudo, Japan; 2 Research Institute of Oral Science, Nihon University School of Dentistry at MatsudoMatsudo, Japan

**Keywords:** interleukin-1β, macrophage inflammatory protein-3α, synovial fibroblast-like cell, temporomandibular joint, tumor necrosis factor-α

## Abstract

**Background:**

Interleukin-1β (IL-1β) and tumor necrosis factor-α (TNF-α) are key mediators of the intracapsular pathological conditions of the temporomandibular joint (TMJ). Therefore, the gene expression profiles in synovial fibroblast-like cells (SFCs) from patients with internal derangement of the TMJ were examined after they were stimulated with IL-1β or TNF-α to determine which genes were altered.

**Methods:**

Ribonucleic acid was isolated from SFCs after IL-1β or TNF-α treatment. Gene expression profiling was performed using oligonucleotide microarray analysis. On the basis of the results of this assay, we investigated the kinetics of macrophage inflammatory protein-3α (MIP-3α) gene expression using PCR, and protein production in TMJ SFCs stimulated by IL-1β or TNF-α using an ELISA. Inhibition experiments were performed with MAPK and NFκB inhibitors. SFCs were stimulated with IL-1β or TNF-α after treatment with inhibitors. The MIP-3α levels were measured using an ELISA.

**Results:**

Macrophage inflammatory protein-3α was the gene most upregulated by IL-1β- or TNF-α stimulation. The mRNA and protein levels of MIP-3α increased in response to IL-1β in a time-dependent manner. In contrast, during TNF-α stimulation, the MIP-3α mRNA levels peaked at 4 h, and the protein levels peaked at 8 h. In addition, the IL-1β- and TNF-α-stimulated MIP-3α production was potently reduced by the MAPK and NFκB signaling pathway inhibitors.

**Conclusion:**

Interleukin-1β and TNF-α increased the MIP-3α production in SFCs *via* the MAPK and NFκB pathways. These results suggest that the production of MIP-3α from stimulation with IL-1β or TNF-α is one factor associated with the inflammatory progression of the internal derangement of the TMJ.

## Introduction

Intracapsular pathological conditions of the temporomandibular joint (TMJ), such as disc displacement (DD), internal derangement (ID), and/or osteoarthritis (OA), tend to cause arthralgia with restriction of the mandibular motion. Synovitis, which frequently accompanies TMJ ID and/or OA, is characterized by chronic inflammatory changes, such as hyperplasia of the synovial lining 1 and an increased number of new capillaries and small vessels 2, with subsequent inflammatory cell infiltration around blood vessels 3–5. Synovial fibroblasts produce a number of putative mediators of inflammation, including cytokines 6.

Interleukin-1β (IL-1β) is a proinflammatory cytokine, with an elevated expression in the joints with ID known to result in the activation of the inflammatory and degradative pathways in synovial cells. Our previous study, using an oligonucleotide microarray analysis, demonstrated the gene expression profiles of IL-1β-stimulated synovial fibroblast-like cells (SFCs) from patients with ID of the TMJ 7. Like IL-1β, tumor necrosis factor-α (TNF-α) is a potent multifunctional cytokine involved in the host immune and inflammatory responses. IL-1β and TNF-α are recognized contributors to the pathogenesis of joint diseases like rheumatoid arthritis (RA), thus leading to synovial fibroblast hyperplasia and the destruction of the extracellular matrix 8–10. Previous studies have also shown appreciable amounts of IL-1β and TNF-α in the synovial fluid of patients with ID of the TMJ 11–14.

In this study, an oligonucleotide microarray analysis in IL-1β- or TNF-α-stimulated SFCs was performed. Macrophage inflammatory protein-3α (MIP-3α) was the most upregulated gene by both IL-1β and TNF-α. MIP-3α, also known as CCL20, or liver and activation regulated chemokine, is a CC chemokine that was identified in 1997 15. Chemokines are small proinflammatory peptides (6-14 kDa) whose main biological function is to recruit certain leukocyte populations to localized sites of inflammation 16. One report showed MIP-3α to be elevated in the synovium and synovial fluid of RA patients and this elevated level could potentially correlate with the development of the disease 17.

To understand how IL-1β and TNF-α may contribute to the pathological conditions of TMJ, we performed gene expression profiling of IL-1β- or TNF-α-stimulated SFCs, and investigated the effects of such IL-1β and TNF-α stimulation on MIP-3α gene expression and production in SFCs from TMJ patients.

## Materials and methods

### Isolation and culture of synovial fibroblast-like cells

Human synovial tissue was obtained from patients who underwent TMJ arthroscopy for ID (TMJ1-6; six females; age range 18-25 years; no other diseases; TMJ1-3 were used for the oligonucleotide microarray analysis, TMJ4 for endpoint PCR and real-time PCR, TMJ5 for ELISA, and TMJ6 for studies regarding the inhibition of ERK, p38, JNK, and NFκB). All patients provided informed consent for the surgery and for the use of their tissue specimens for research purposes. The isolation, primary culture, and experimentation with synoviocytes were performed according to the guidelines established by the Institutional Review Board of the Nihon University School of Dentistry at Matsudo, Japan. The ethics committee recognition numbers were EC03–003 and EC07–004.

Synovial fibroblast-like cells from the TMJ patients were prepared using the outgrowth method previously reported by Ogura et al. 18. In brief, tissue samples were washed with phosphate-buffered saline (PBS), minced, placed in a 35-mm tissue culture dish, and covered with a sterilized glass coverslip. The culture medium used was Ham’s F12 (Gibco, Grand Island, NY, USA) supplemented with 10% fetal calf serum (FCS) (Cell Culture Technologies, Gravesano, Switzerland), 100 μg/ml penicillin G (Meiji, Tokyo, Japan), 100 μg/ml kanamycin sulfate (Meiji), and 250 ng/ml fungizone (Gibco). The medium was changed twice per week. Confluent SFCs were detached with 0.025% trypsin (Gibco) and 0.02% EDTA in PBS, and then subcultured in Ham’s F12 supplemented with 10% FCS and antibiotics. The cells isolated from the TMJ synovium were examined for cell markers of myofibroblasts 19. For the experiments, we used SFCs obtained from passages 6 to 8.

### Total RNA extraction

Synovial fibroblast-like cells were plated at 1 × 10^6^ cells per 100-mm dish in Ham’s F12 medium containing 10% FCS and antibiotics. Confluent cells were cultured for 24 h in the same medium containing 2% FCS, and then were incubated with or without IL-1β (0.1 ng/ml) or TNF-α (10 ng/ml) for 2, 4, and 8 h. The cells were harvested and homogenized with 1 ml TRIZOL reagent (Life Technologies, Gaithersburg, MD, USA) with a FastPrep FP120 homogenizer (BIO 101, Vista, VA, USA). Total RNA was extracted using the Acid Guanidinium Thiocyanate-Phenol-Chloroform Extraction (AGPC) method 20. Briefly, 200 μl of chloroform was added to homogenized tissue samples. The aqueous phase was transferred to a new tube, and chloroform was then added to the homogenate. This aqueous phase was transferred to a new tube, and phenol chloroform isoamylalcohol (24:25:1) was added. The aqueous phase was then transferred to a new tube, and chloroform was added again. The aqueous phase was transferred to a new tube, and isopropanol was added to precipitate the total cellular RNA, which was stored in ethanol at −80°C until use.

### Oligonucleotide microarray analysis

Total RNA samples from SFCs treated with IL-1β (0.1 ng/ml) or TNF-α (10 ng/ml) for 4 h and untreated control samples were run on an RNA 6000 Nano Gel System (Agilent Technologies Inc., Santa Clara, CA, USA) using the Agilent 2100 Bioanalyzer (Agilent) for RNA quality determination. Total RNA samples (TMJ 1, 2, 3, controls, IL-1β-treated and TNF-α-treated) were profiled on a Human Genome Focus Array (Affymetrix, Santa Clara, CA, USA), according to the manufacturer’s instructions.

Raw data from nine GeneChips were loaded into the GeneSpring software program (version 11; Agilent Technologies, Waldbronn, Germany). The data were normalized using the median of raw data from each array as a reference, and then were analyzed.

Biologically relevant pathways were constructed using the Ingenuity Pathway Knowledge Base (IPA) (Ingenuity, Redwood, CA, USA). The gene accession numbers and gene expression ratios (IL-1β treated/control) or (TNF-α/control) of greater than 2-fold intensity as determined by the GeneChip software program were upload into the IPA. These genes, known as focus genes, were used as the starting point to generate the biological network.

### Endpoint PCR

Complementary DNA was synthesized, and amplifications were performed using a GeneAmp RNA PCR kit (Perkin-Elmer, Norwalk, CT, USA). Amplification of the PCR mixture was performed with the GeneAmp PCR system 9600 (Perkin-Elmer), beginning with pre-heating at 94°C for 5 min, followed by 19 cycles as follows: 94°C for 1 min, 55°C for 2 min, and 72°C for 2 min. PCR fragments were electrophoresed on 1.5% agarose gels, followed by staining with ethidium bromide and examination of fragment sizes.

The primers for MIP-3α were F: 5′-GCA AGC AAC TTT GAC TGC TG-3′and R: 5′-CAA GTC CAG TGA GGC ACA AA-3′; the PCR product obtained with these primers was 342 bp in size. The primers for GAPDH, used to normalize the MIP-3α expression, were F: 5′-ATC ACC ATC TTC CAG GAG-3′ and R: 5′-ATC GAC TGT GGT CAT GAG-3′; the PCR product was 315 bp in size.

### Real-time PCR

The cDNA was again synthesized using a GeneAmp RNA PCR kit (Perkin-Elmer). Real-time PCR was performed using a DyNAmo SYBR green qPCR kit (Finnzymes, Espoo, Finland). The PCR mixture (20 μl) contained 20 pmol forward and reverse primers and 2 μl cDNA. Amplification was performed using a DNA Engine Opticon 1 (Bio-Rad, Hercules, CA, USA), with pre-heating at 95°C for 10 min, followed by 40 cycles as follows: 94°C for 15 s, 57°C for 30 s, and 72°C for 30 s. Amplicons were detected directly by measuring the increase in fluorescence caused by the binding of SYBR Green I dye to gene-specific, amplified, double-stranded DNA. Following the completion of PCR amplification, the temperature was increased from the annealing temperature to 95°C for a melting curve analysis. The real-time PCR experiment was independently performed three times.

The initial template concentration was derived from the cycle number at which the fluorescent signal crossed the threshold cycles of MIP-3α and GAPDH. ΔC_T_ (C_T_-MIP-3α minus C_T_-GAPDH) indicates the relative amount of MIP-3α transcripts. ΔΔC_T_ (ΔC_T_-treated minus ΔC_T_-control) represents the relative *n*-value compared with the control. The quantity 2^−*n*^ represents the difference in MIP-3α expression between the IL-1β- or TNF-α-stimulated cells and the controls.

### MIP-3α enzyme-linked immunosorbent assay

Synovial fibroblast-like cells were plated at 5 × 10^4^ cells per well in 24-well plates with Ham’s F12 medium containing 10% FCS. Confluent cells were cultured for 24 h in the same medium containing 2% FCS. After incubation with IL-1β or TNF-α for the appropriate length of time, culture supernatants were collected and stored at −80°C until use. We examined the kinetics of MIP-3α protein production in control samples and synovial fibroblasts incubated with IL-1β (0.1 ng/ml) or TNF-α (10 ng/ml) for 4, 8, 24, and 48 h. To examine the dose dependency of MIP-3α protein expression, the cells were treated with IL-1β at concentrations ranging from 0.001 to 1 ng/ml and with TNF-α at concentrations ranging from 0.001 to 1 ng/ml for 24 h. The MIP-3α levels in conditioned medium were measured using an ELISA kit (R&D Systems, McKinley, MN, USA), according to the manufacturer’s protocol. The ELISA experiments were independently performed four times.

### Inhibition of ERK, p38, JNK, and NFκB

Synovial fibroblast-like cells were plated at 5 × 10^4^ cells per well in 24-well plates with Ham’s F12 medium containing 10% FCS. Confluent cells were cultured for 24 h in medium containing 2% FCS. The inhibition experiments were performed with PD98059 (ERK1/2 inhibitor: 40 μM) (Alexis Biochemicals, San Diego, CA, USA), SB203580 (p38 inhibitor: 10 μM) (Alexis Biochemicals), SP600125 (JNK1/2 inhibitor: 10 μM) (Biomol, Plymouth Meeting, PA, USA), or ammonium pyrrolidine dithiocarbamate (APDC) (NFκB inhibitor: 10 μM) (Calbiochem, San Diego, CA, USA). The cells were pre-treated with these reagents for 15 min, followed by incubation with IL-1β (0.1 ng/ml) or TNF-α (10 ng/ml). The control for the inhibitor experiments was synovial fibroblasts treated with IL-1β or TNF-α without inhibitors. After 4 h, the culture supernatants were collected and stored at −80°C until use. The inhibitor effect was calculated as: (MIP-3α production with IL-1β or TNF-α)/(MIP-3α production with IL-1β or TNF-α in the presence of the inhibitor). The MIP-3α levels in the conditioned medium were measured using an ELISA kit (R&D Systems).

### Statistical analysis

We assayed the real-time PCR in triplicate and performed ELISA using four replicates. The data are expressed as the mean values ± SD. Differences between the MIP-3α expression in the control cells and in the cells treated with IL-1β or TNF-α were calculated using Student’s *t*-test. The statistical significance for multiple comparisons was assessed using one-way ANOVA.

## Results

### Evaluation of the MIP-3α mRNA expression by GeneChip arrays

The expression of 8793 genes on the Human Genome Focus Array in control and IL-1β- or TNF-α-stimulated cells was compared. A total of 212 genes showed a greater than 2-fold upregulation by IL-1β, while 239 genes were upregulated at least 2-fold by TNF-α. Table 1 lists the 10 most upregulated genes by IL-1β and TNF-α. There were five genes that overlapped for the two treatments, and MIP-3α was found to be the most highly upregulated gene by both IL-1β and TNF-α (Table 1). In fact, approximately 50% of the top 10 genes upregulated by IL-1β and TNF-α, respectively, were chemokines (Table 1).

**Table 1:** Up regulated genes by treatment with IL-1β or TNF-α

	IL-lβ			TNF-α		
Rank	Gene	GenBank ID	Fold	Gene	GenBank ID	Fold
1	CCL20 (MIP-3α)	NM_004591	429.9	CCL20 (MIP-3α)	NM_004591	322.2
2	CXCL3 (GRO-γ)	NM_002090	150.4	IL-8 (CXCL8)	AF043337	76.7
3	CSF2	M11734	107.4	CSF2	M11734	37.9
4	IL-8 (CXCL8)	AF043337	89.8	ICAM1	NM_000201	32.1
5	CXCL1 (GRO-α)	NM_001511	59.5	CXCL3 (GRO-γ)	NM_002090	31.1
6	CXCL2 (GRO-β)	M57731	50.1	CXCL10 (IP 10)	NM_001565	27.8
7	IL-6	NM_000600	40.1	BCL2A1	NM_004049	24.5
8	PTGS2 (COX-2)	NM_000963	37.8	GCH1	NM_000161	21.9
9	BCL2A1	NM_004049	37.3	IL17RB	NM_019583	21.9
10	CXCL10 (IP10)	NM_001565	28.7	CX3CL1 (flactalkine)	U84487	21.6

Rank: ranking of up regulated gene by IL-β or TNF-α. Fold: average normalized intensity of stimulated SFC in TMJ1-3/average normalized intensity of control SFC in TMJ1-3.

### MIP-3α gene expression

**Figure 1:**
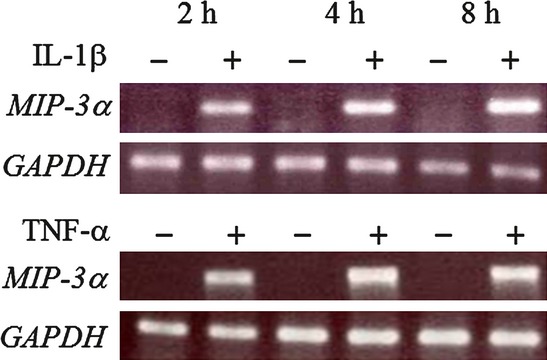
Agarose gel depicting relative MIP-3α mRNA levels in synovial fibroblast-like cells treated with IL-1β or TNF-α by endpoint PCR. Cells were either left untreated or treated with 0.1 ng/ml IL-1β or 10 ng/ml TNF-α for 2, 4, or 8 h. GAPDH was analyzed as an internal control.

**Figure 2:**
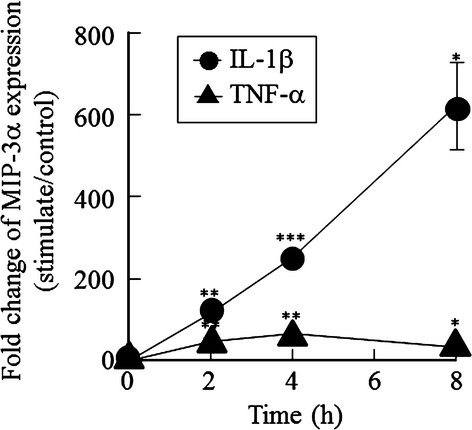
Real-time PCR analysis of the MIP-3α mRNA levels in synovial fibroblast-like cells treated with IL-1β or TNF-α. The cells were treated with 0.1 ng/ml IL-1β or 10 ng/ml TNF-α for 2, 4, or 8 h. Fold changes were calculated using the ΔΔCT method. Mean ± SD (*n* = 3). *P < 0.05, **P < 0.01, ***P < 0.005 compared with the untreated control cells.

As MIP-3α was the most highly upregulated gene by both treatments, we examined the kinetics of MIP-3α gene expression in SFCs stimulated with IL-1β or TNF-α. The MIP-3α mRNA levels in SFCs were elevated time dependently by IL-1β stimulation; following TNF-α stimulation, they were increased at 2 h and 4 h, and then decreased after 8 h (Figure 1). The results of the real-time PCR analysis were similar to those of endpoint PCR; the MIP-3α mRNA levels increased with IL-1β stimulation in a time-dependent manner, while the mRNA levels peaked at 4 h in the cells stimulated with TNF-α (Figure 2).

### MIP-3α protein levels

**Figure 3:**
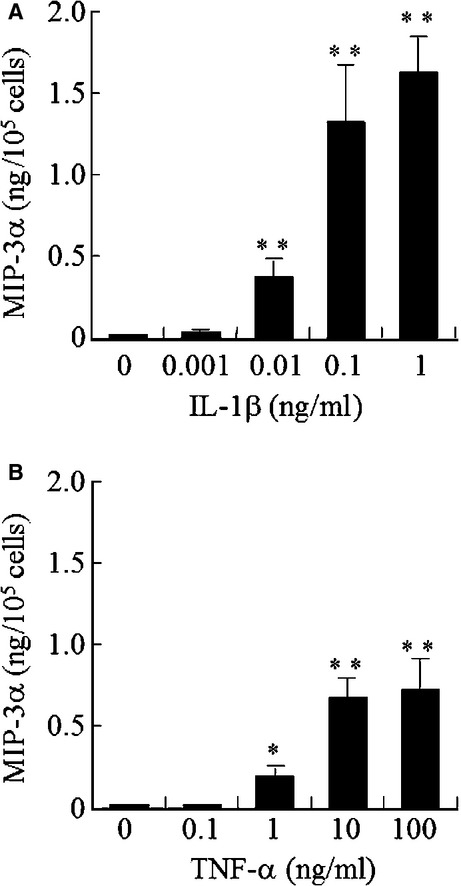
Effects of IL-1β and TNF-α on the MIP-3α protein levels in conditioned medium from human TMJ synovial fibroblast-like cells. The cells were stimulated with the indicated concentrations of (A) IL-1β or (B) TNF-α for 24 h, and the MIP-3α protein levels in the conditioned medium were then assayed using an ELISA. Mean ± SD (*n* = 4 replicates). * *P* < 0.05, **P < 0.005, compared with the untreated control cells. Significant differences (*P* < 0.05) in IL-1β and TNF-α were determined using one-way ANOVA.

**Figure 4:**
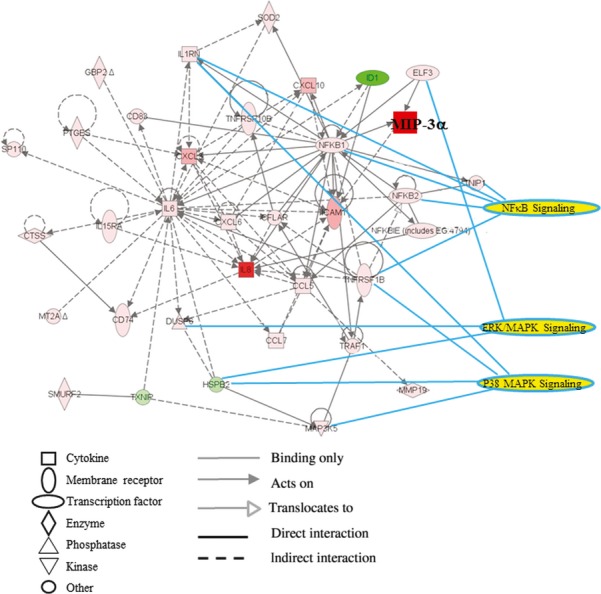
Enzyme-linked immunosorbent assay of MIP-3α protein levels in conditioned medium from human TMJ synovial fibroblast-like cells over time with cytokine treatment. Cells were treated with 0.1 ng/ml IL-1β or 10 ng/ml TNF-α for 4, 8, 24, or 48 h. Mean ± SD (*n* = 4). *P < 0.05, **P < 0.005 compared with the untreated control cells.

Synovial fibroblast-like cells were incubated with concentrations of IL-1β ranging from 0.001 to 1 ng/ml for 24 h. IL-1β increased the MIP-3α production in a dose-dependent manner, although there was no significant difference between the cells treated with 0.001 ng/ml IL-1β and the untreated controls (Figure 3A). Next, SFCs were incubated for 24 h with concentrations of TNF-α ranging from 0.1 to 100 ng/ml. TNF-α also increased the production of MIP-3α in a dose-dependent manner up to 10 ng/ml, at which concentration the expression plateaued. There was no significant difference between the cells treated with 0.1 ng/ml TNF-α and the untreated control cells (Figure 3B). In the next experiment, we examined the kinetics of MIP-3α protein production in control samples and SFCs incubated with 0.1 ng/ml IL-1β or 10 ng/ml TNF-α for 4, 8, 24, and 48 h. The MIP-3α protein production was stimulated by IL-1β in a time-dependent manner over the entire 48-h period, whereas the TNF-α-mediated stimulation peaked at 8 h and then plateaued (Figure 4).

### Effect of inhibitors on MIP-3α production

**Figure 5:**
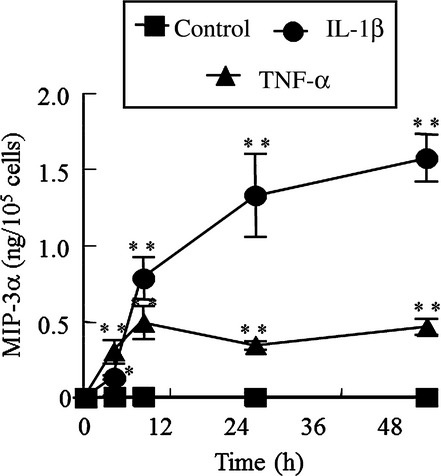
Network of IL-1β- and/or TNF-α-induced molecules by IPA. Data were analyzed using Ingenuity Pathway Analysis (Ingenuity®System, www.ingenuity.com). The intensity of the node color indicates the degree of up- (red) or down- (green) regulation. Nodes are indicated by various shapes that represent the functional class of the gene product. The lines are displayed with various labels that describe the nature of the relationship between the nodes.

To investigate the IL-1β or TNF-α signaling pathway involved in MIP-3α production in SFCs, we uploaded the genes of stimulated cells that showed a greater than 2-fold increase in intensity by the GeneChip analysis compared with controls into the IPA as focus genes. This gene subset was arranged into nine molecular networks, as defined by the IPA. MIP-3α was a component of Networks 1 (data not shown). MIP-3α and NFκB were upregulated by IL-1β or TNF-α in SFCs. These networks were linked in a graphical representation of the canonical pathways “MAPKs signaling” and “NFκB signaling” in the IPA (Figure 5). This result indicated that IL-1β or TNF-α induced the activation of p38MAPK-, JNK-, and NFκB-mediated TAK1 activation. We therefore tried to elucidate whether the NFκB and MAPK pathways are required for the induction of MIP-3α by IL-1β or TNF-α in SFCs by performing an analysis of the effect of the MAPK inhibitors, PD98059, SB203580, and SP600125, and the NFκB inhibitor, APDC, on such induction. We found that MIP-3α production was inhibited by more than 63% by PD98059, SB203580, SP600125, and APDC treatment in both IL-1β- and TNF-α-stimulated cells (Table 2).

**Table 2:** Effect of inhibitor on MIP-3α production

	IL-1β		TNF-α	
Inhibitor	MIP-3α (pg/10^5^ cells)	% inhibition	MIP-3α (pg/10^5^ cells)	% inhibition
None	118.0 ± 27.2	0	482.1 ± 223.2	0
PD98059 (ERK1/2)	43.8 ± 15.2	63	142.7 ± 23.2	70
SB203580 (p38)	I9.7 ± 4.0	83	115.8 ± 17.9	76
SP600125 (JNK)	14.6 ± 8.5	88	80.8 ± 11.9	83
APDC (NFκB)	15.3 ± 1.9	87	17.2 ± 3.8	96

The SFCs were pre-treated with 40 µM PD98059, 10 µM SB203580, 10 µM SP600125 or 10 µM APDC for 15 min, and then treated with 0.1 ng/ml IL-1β or 10 ng/ml TNF-α for 3 h. Results are expressed as means ± SD (*n* = 4).

## Discussion

Interleukin-1β and TNF-α play important roles as proinflammatory cytokines involved in ID 11–14. In this study, we examined the gene and protein expressions of MIP-3α in SFCs derived from TMJ patients in response to treatment with IL-1β or TNF-α because MIP-3α was found to be the most highly upregulated gene in SFCs by IL-1β and TNF-α microarray analysis. The effects of IL-1β on MIP-3α protein expression were longer lasting than those of TNF-α. MIP-3α has a chemoattractant effect on CCR6 leukocytes, such as immature dendritic cells, memory T cells, and naive B cells, all of which express its receptor 21–23. It is well known that RA synovial tissues contain many CCR6-expressing leukocytes 24–26, and MIP-3α and CCR6 have been detected in the synovial fluid and synovia from RA patients 27.

A previous study demonstrated that the nucleotide sequence of the human MIP-3α promoter region has binding sites for Ets, AP-1, SP-1, and NFκB 28. Other reports have described that ERK, p38, and NFκB play an important role in mediating the production of MIP-3α induced by IL-1β in gingival fibroblasts and airway epithelial cells 29, 30. Ets activates ERK1/2 31, p38 MAPK activates the transcription factor SP-1 32, and JNK activates c-Jun, a component of the AP-1 transcription factors 33. PD98059 is a specific inhibitor that binds to the inactive forms of MAPK/ERK kinase (MEK) and prevents their activation and phosphorylation, thus resulting in the inhibition of ERK 34. SB203582 is a selective inhibitor of p38 MAPK that inhibits the activation of MAPKAP K2, a specific physiological substrate of p38 MAPK 35. SP600125 inhibits the phosphorylation of JNK through competitive binding to the JNK ATP-binding site 36. APDC is an antioxidant that can block the activation of NFκB by inhibiting IκB degradation 37. In this study, pre-treatment of synovial cells with PD98059, SB203580, SP600125, or APDC inhibited the induction of MIP-3α protein production by both IL-1β and TNF-α. These data suggest that the induction of MIP-3α production by IL-1β or TNF-α occurs through ERK, p38 MAPK, JNK, and NFκB activation in SFCs derived from the TMJ.

Inflammatory cells have been detected in synovial tissues from TMJ ID patients 38. Inflammatory cells produce cytokines, matrix metalloproteinases (MMPs), and reactive oxygen species (ROS) in RA 39, 40. The accumulation of inflammatory cells in synovial tissues may lead to the degradation of this tissue in joints through the production of MMPs and ROS 41. This study demonstrated that MIP-3α production was induced by IL-1β or TNF-α through the ERK, p38 MAPK, JNK, and NFκB pathways in human SFCs. Increased MIP-3α may trigger the migration of dendritic cells, T cells, and B cells into the synovial tissue and fluid of TMJ ID patients, and may cause the initiation and progression of inflammatory changes in the TMJ. The migration of CCR6-expressing leukocytes has been reported to decrease by approximately 70% following treatment with an anti-MIP-3α antibody *in vivo* and *in vitro* 9, 23. Anti-chemokine therapy has been investigated as a possible new approach in RA patients 42, 43. The new anti-rheumatic drugs KE-298 and epigallocatechin-3-gallate decrease the production of chemokines in RA synovial fibroblasts 44, 45. Therefore, the use of anti-MIP-3α therapy may become important as a possible new interventional approach for RA. Similarly, understanding the mechanisms of IL-1β and TNF-α signaling could provide new therapeutic approaches for preventing the activation of inflammatory processes in the TMJ.

Currently, conservative therapies, such as splinting and physical therapy, are the main treatments for ID patients. We have recently performed a few surgical procedures for ID of the TMJ 46. This study was limited by the difficulty of obtaining synovial fibroblasts in sufficient quantities, as the TMJ is a small joint space in comparison with other joints (shoulder, knee, and hip). We have therefore performed only a few surgical procedures for ID/OA of the TMJ.

In conclusion, we isolated SFCs from diseased human TMJs, and examined how their response to stimulation with IL-1β or TNF-α affects the underlying inflammatory status of this joint. The excessive production of MIP-3α in SFCs stimulated by IL-1β or TNF-α through the NFκB and MAPK pathways may be related to the pathological conditions of the TMJ. These findings may help define new therapeutic targets for the inflammatory components of ID and OA. We consider that further studies of both the cellular and molecular mechanisms are necessary to improve the diagnosis of, and therapy for, pathological conditions of the TMJ.
